# Development and evaluation of an escape room based on general pharmacokinetics: Students' perceptions of its motivational climate

**DOI:** 10.1002/prp2.1155

**Published:** 2023-11-29

**Authors:** Anneke van Houwelingen, Freija ter Heegde, Wendy Boschloo, Leonie Piek, Theo Wubbels

**Affiliations:** ^1^ Department of Pharmaceutical Sciences, Science Faculty Utrecht University Utrecht The Netherlands; ^2^ Department of Education and Pedagogy, Faculty of Social and Behavioural Sciences Utrecht University Utrecht The Netherlands

**Keywords:** escape room, mixed‐method design, motivational climate, pharmacokinetics, undergraduate

## Abstract

We designed an escape room based on the basic principles of pharmacokinetics for undergraduate bachelor students and explored its effect on students' perceived motivational climate and usefulness as a formative assessment via a mixed‐method design. The effect on students' perceptions of the motivational climate was measured using pre‐ and post‐test measurements of the MUSIC® inventory. Students' experiences with the escape room and suggestions for improvement were collected by open‐ended survey questions. Forty‐one students initially joined the study while 28 students completed both the pre‐ and post‐test MUSIC® inventory. Data from the MUSIC® inventory revealed the effect of playing the escape room on students' situational interest was positive with medium to large effect (Cohen's *d*_av_ = 0.63). Data from the open‐ended questions confirmed the outcome of the MUSIC® inventory. While there was a positive effect on situational interest, students found the escape room not very useful as a tool for formative assessment. Further research should include a control group and focus on the effect of the escape room on academic success and work toward increasing the capacity of the escape room for large‐scale courses.

## Graphical abstract

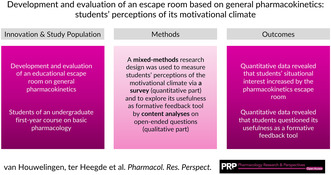

**Abbreviation**
CPS: College of Pharmaceutical Sciences

## 1 INTRODUCTION

It is well known that the more students are motivated and engaged in their learning process, the better they do in higher education as measured by test performances, well‐being scores, and dropout intention.^1^, ^2^, ^3^ Programs or courses that stimulate students' engagement and motivation need to both implement motivating and engaging tools and techniques and have teachers who help students take responsibility for their personal learning journey.^2^, ^4^, ^5^

As recently demonstrated, Dutch health care and life science students experience difficulties with concepts of general pharmacology, especially pharmacokinetics.^6^, ^7^, ^8^ Not only did students experience difficulties with pharmacokinetics in general, students also scored lower on higher order thinking questions related to the pharmacokinetics compared to those on pharmacodynamics.^7^ Although pharmacy students had better knowledge of basic pharmacological principles compared to medical students, there is still room for improvement.^8^ These findings suggest a need for a different approach to teaching general pharmacology to undergraduate students.

One way to enhance students' performance on pharmacokinetics is strengthening students' engagement and motivation by introducing game‐based learning. Different game‐based learning experiences (e.g., an escape room) in the pharmaceutical context could improve students' engagement, motivation, and test scores.^9^ In pharmacy education, escape rooms are typically built around a specific disease, therapeutical interventions, or focused on the development of skills.^9^, ^10^, ^11^, ^12^, ^13^, ^14^ Escape rooms dealing solely with general pharmacokinetics are scarce. We have, therefore, developed an escape room with puzzles and quizzes based on the basic principles of the pharmacokinetics.

In this study, the MUSIC® model for academic motivation was used to measure students' self‐perception of the motivational climate of the escape room. This model was developed to evaluate students' perceptions of the motivational climate in five different categories: eMpowerment, Usefulness, Success, Interest, and Caring.^15^ By tapping into one or more of these categories, teachers and educators can improve the motivational climate of their courses and course activities with relatively simple adjustments, thereby enhancing students' engagement and motivation.^5^

The aim of this study was to design, implement, and explore the effect of a general pharmacokinetics‐based escape room on the students' perceptions of its motivational climate through the lens of the MUSIC® model. Additionally, students' experiences of the escape room as a formative assessment were evaluated by two open‐ended questions.

## 2 METHODS

### 2.1 Course setting and design of the escape room

The escape room was developed as part of a first‐year compulsory course on general pharmacology of the undergraduate program of the College of Pharmaceutical Sciences (CPS). This selective and international honors program focuses on drug development and drug research.^16^ The pharmacology course was taught in the second part of the first semester. It had a study load of 400 h, yielding 15 credits according to the European Credit Transfer System. The pharmacology course covered both general pharmacokinetics and pharmacodynamics.

The escape room was designed by a co‐creation development team of two honors students and two honors lecturers. The overall goal of the escape room was to practice the core principles of absorption, distribution, metabolism, and excretion of drugs in a playful interactive environment. Once the overall workflow of the escape room had been developed, puzzles and quizzes were made in alignment with the learning goals of the course.

**FIGURE 1:**
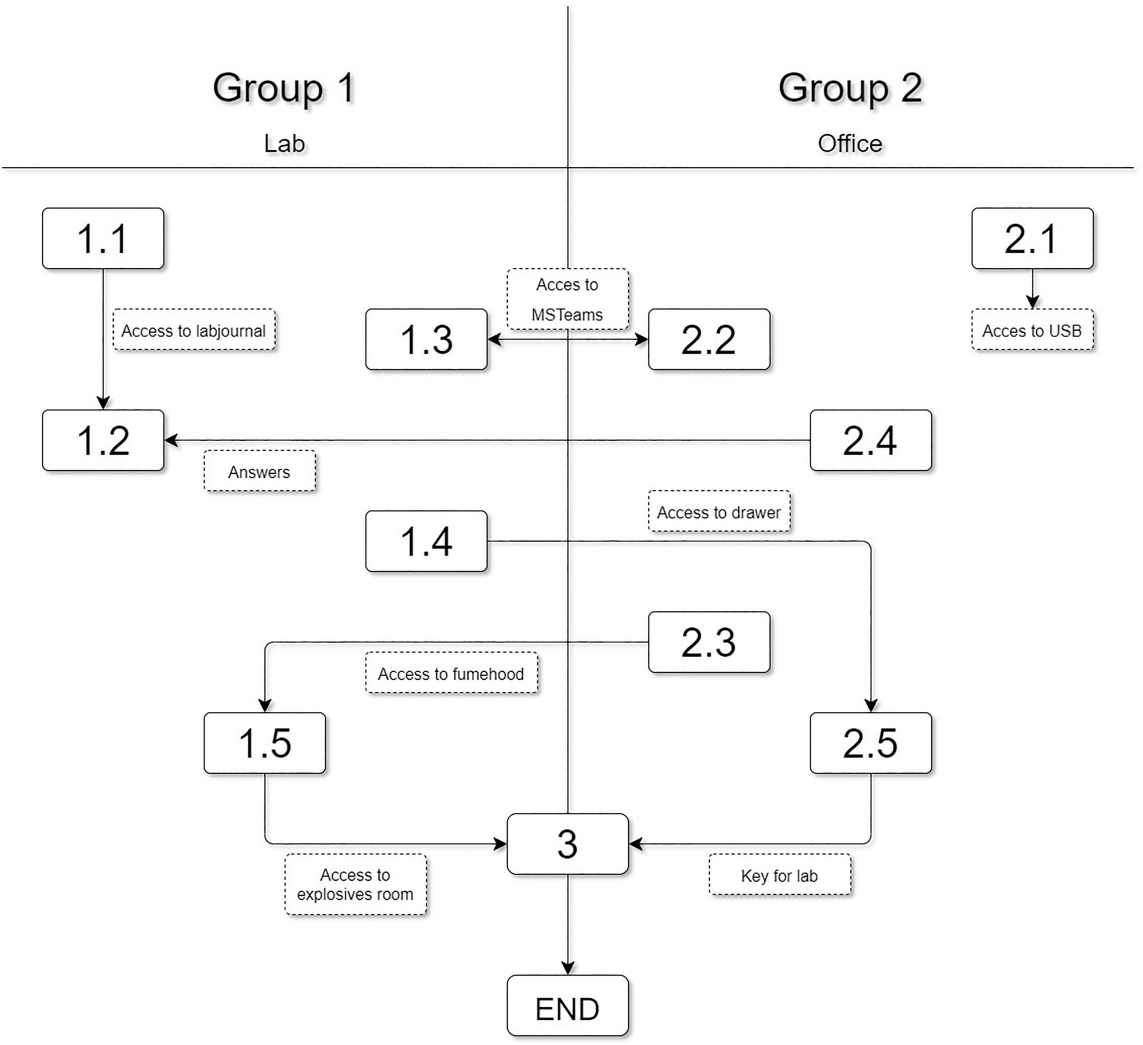
General overview of the flowchart of the pharmacokinetics escape room. Numbers refer to the tasks and descriptions of Table 1.

**FIGURE 2:**
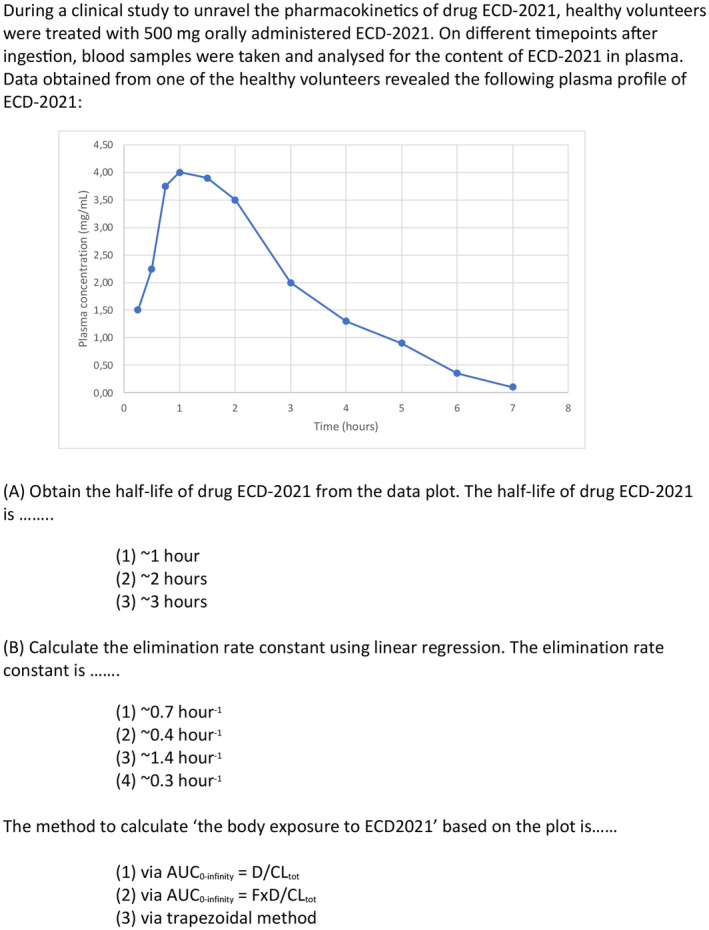
An example puzzle that students encountered while playing the escape room.

During the escape room exercise, students had to work together to defuse an imaginary bomb made by a scientist who was originally a respected drug researcher involved in the development of innovative drug delivery systems for nitroglycerin. Two groups of maximum three students each, started on campus in different locations each equipped with a computer and internet facilities. At the start, the groups did not know that they had to work together, but as the game progressed, they were connected via a Microsoft TEAMS using the computer and internet facilities. By exchanging information obtained from solving puzzles and quizzes the groups had to work together to obtain the final location of the bomb and defuse the bomb with one final puzzle consisting of four questions. The total game time was set at maximum 90 min. Directly after the game, there was a debriefing session to blow off steam and ask questions related to the puzzles and quizzes students encountered. In‐depth information on the processes and actions during the game are depicted in Table 1 while a simple flowchart of the game is depicted in Figure 1. An example puzzle is found in Figure 2.

**TABLE 1:** Overview of tasks students encounter during the escape room.

	Task	Description
Group 1	1.1	Solve puzzle to get location of the lab book, discover by reading through Mr. Simon's notes that he is making an explosive
	1.2	Read through lab book, realize that answers on puzzles should be with group 2, and therefore find a way to communicate with group 2
	1.3	Find laptop and solve puzzle to get access to the laptop
	1.4	Find and solve the next puzzle on laptop by scrolling through documents and files on the laptop, answers are necessary for group 2 to get access to a locked drawer
	1.2/2.4	Communication between groups 1 and 2, share information to solve puzzles
	1.5	Solve puzzles, solutions indicate the location of the explosive and a code word to get beyond the gatekeeper of the bomb (a game assistant)
	3	Wait for group 2 to unlock the lab
Group 2	2.1	Solve puzzle on top of the desk to get access to USB
	2.2	Find laptop and solve puzzle to get access to the laptop, plug in USB device and watch video in which Mr. Simon explains why he is making an explosive.
	2.3	Find and solve the next puzzle on laptop by scrolling through documents and files on the laptop, answers are necessary for group 1
	2.4/1.2	Communication between groups 1 and 2, share information to solve puzzles
	2.5	Solve puzzles, solutions indicate the location of group 1 and a code word (e.g. key for lab) to ‘unlock’ group 1 from the gatekeeper of the classroom (a game assistant)
	3	Move to the location of group 1 and unlock the lab
Groups 1 and 2	3.1	Move to the next location
	3.2	Open the door of the location, timer begins ticking (max. 5 min)
	3.3	Solve multiple‐choice puzzles to defuse the bomb, there is a neutralizer possibility in case of one wrong answer
	3.4	Puzzles solved correctly before timer is done, explosive defused, game won
		If more than one answer wrong or timer expires, explosion, and game lost

Before implementing the escape room in the course on pharmacology of the academic year 2021–2022, it was piloted in October 2021 with a small number of second‐year CPS students that already had passed the course.

### 2.2 Study design

Students of the course on basic pharmacology were invited to join this mixed‐method study. For this, students were asked to complete the MUSIC® inventory pre‐ and post‐intervention. Students could choose either the escape room (intervention) or an online digital test (control) as a way of formative testing. Puzzles and quizzes that students encountered during online testing were identical to those used in the escape room. Forty‐one students (68%) gave their informed consent. From these, 30 students completed both pre‐ and post‐inventory. Because only two students in the control group completed both pre‐ and post‐test inventory it was decided to focus only on the data of the intervention group.

### 2.3 Measures

Students were asked to complete the MUSIC® inventories via Qualtrics during an on‐campus classroom activity 3 days before the escape room (pre‐test). The post‐test inventory was completed directly after the online test (control group) or during the debriefing session of the escape room (intervention group).

The MUSIC® inventory is a 26‐item 6‐point Likert scale questionnaire based on five scales: eMpowerment, Usefulness, Success, Interest, and Caring. This inventory was developed by Jones,^15^ from whom the first author got permission to use the inventory. Coefficient alpha showed adequate reliability for eMpowerment (α = 0.79 [pre‐test]; α = 0.85 [post‐test]), Usefulness (α = 0.83 [pre‐test]; α = 0.87 [post‐test]), Success (α = 0.88 [pre‐test]; α = 0.75 [post‐test], Interest (α = 0.83 [pre‐test]; α = 0.83 [post‐test]), and Caring (α = 0.89 [pre‐test]; α = 0.91 [post‐test]). The post‐test MUSIC® inventory was accompanied by two open‐ended questions; “Do you have any general comments regarding this format of formative testing?” (Q1) and “What could be improved for next year?” (Q2).

### 2.4 Data analysis

Data from the MUSIC® inventory was analyzed using Jamovi (version 2.3.0.0) and SPSS (version 28). The number of missing data was very limited and completely random. Therefore, the average of the scales was calculated by list‐wise deletion of missing values. Repeated measures multivariate analysis (RM‐MANOVA) was used to establish differences in scores on the MUSIC® inventory between the pre‐ and post‐test data while controlling for multiple comparisons. If significance was detected, average scores of the individual scales were further analyzed by two‐tailed paired samples *t*‐test. Effect sizes (Cohen's *d*_av_) were calculated to investigate the effectiveness of the intervention on students' perception of the motivational climate.

Content analyses were employed on the open‐ended questions.^17^ For this, fixed units of analysis were coded based on the scales of the MUSIC® model by the first author of the manuscript. Units could receive multiple codes. The category ‘Usefulness’ was further subdivided into positive or negative experiences of the escape room as formative assessment.

### 2.5 Nomenclature statement

Key protein targets and ligands in this article are hyperlinked to corresponding entries in https://www.guidetopharmacology.org, the common portal for data from the IUPHAR/BPS Guide to PHARMACOLOGY,^18^ and are permanently archived in the Concise Guide to PHARMACOLOGY 2019/20.^19^

## 3 RESULTS

**FIGURE 3:**
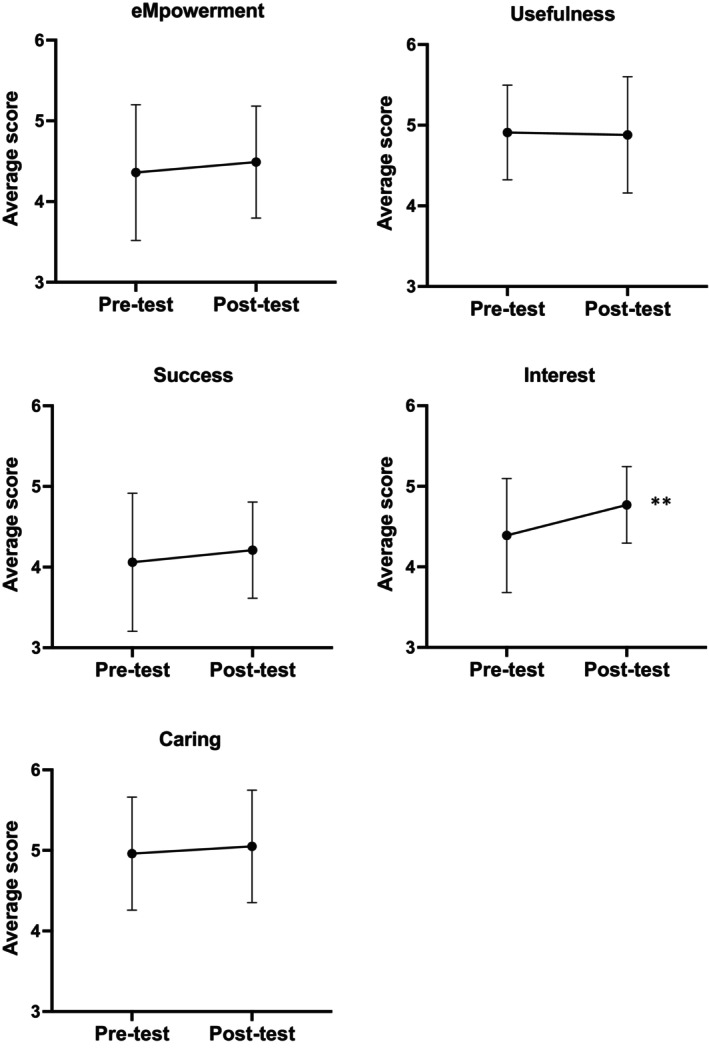
Pre‐ and post‐measures (average score (SD)) of the MUSIC® inventory. Significancy between pre‐ and post‐test data of the intervention group tested by two‐tailed paired *t*‐test. **p < .001.

Pre‐ and post‐measures of the MUSIC® inventory are presented in Figure 3. RM‐MANOVA analysis revealed a significant effect in time in the intervention group, Hotelling's Trace = 0.29, *F*(1,27) = 7.83, *p* = .009, *η* ^2^_*p*_ = 0.23. A paired samples *t*‐test revealed that the mean of the post‐test of the Interest scale was significantly higher than the mean of the pre‐test, *t*(27) = 3.83, *p* < .001, 95% CI Mean_diff_ [0.18, 0.58], Cohen's *d*_av_ = 0.63, 95% CI [0.32, 0.99]. There was no significant difference on eMpowerment, Usefulness, Success, or Caring.

Analysis of the open‐ended question Q1 identified four categories of the MUSIC® model (see Table 2), with Interest as the most prominent category (24 out of 34 responses). In addition to this, the analysis revealed that some students were happy with the freedom of choice for either the escape room or the online testing or liked the collaborative nature of the escape room. Some students (*n* = 8) questioned the relevance of the puzzles and quizzes in relation to the final exam questions on the pharmacokinetics.

**TABLE 2:** Overview of (sub)categories, number of responses, and example quotes from the answers on open‐ended question Q1 (*N* = 34) and Q2 (*N* = 33).

Category	Number of responses Q1	Number of responses Q2	Example quotes
eMpowerment			
Positive experience	2	n.d.	“I think it's nice that students can choose if they want a strictly theoretical or an active formative test since some people learn better if it's tested in an active way.”
Negative experience	n.d.	2	“Maybe a bit more guidance about what to do with certain pieces of information could prove useful when trying to complete the task at hand and escape the room.”
Usefulness escape room			
Positive experience	8	n.d.	“It's a good way to have people actively apply their knowledge, thereby it is also in a playful environment which makes it enjoyable to do.”
Negative experience	10	n.d.	“I really enjoyed it, but it was not exam preparation.”
Improvements	n.d.	17	“More exam level questions and to see answers.”
Success	n.d.	n.d.	n.d.
Interest	24	n.d.	“Very creative and unexpectedly fun.”
Caring	5	2	“I really liked this interactive form and that you had to work together with a group in another room.”
Course improvements	n.d.	8	“Clear explanation of what is compulsory and what is extra information.”

Abbreviation: n.d, equals not determined.

Analysis of the open‐ended question on improvement (Q2) revealed three types of suggestions: (1) suggestions for improvement of the escape room to make it more useful as a formative test (e.g., “More exam level questions and to see answers”), (2) suggestions for improvement of the escape room in general (e.g., “replace one cross word puzzle for a calculation question”), and (3) suggestions for the improvement of the course in general.

## 4 DISCUSSION

The objectives of this study were to develop an escape room and subsequently, implement it and explore students' perceptions of its motivational climate and usefulness as a way of formative testing. After playing the escape room students' situational interest increased as measured by the MUSIC® inventory. This was also illustrated by the outcome of the qualitative analyses where the category Interest was the most prominent category compared to the other categories of the MUSIC® model. The results of this study are in line with other studies that investigated the effects of playful environments, like escape rooms, on motivation and engagement in occupational health therapy students and pharmacy students.^10^, ^11^

It is known that recreational and educational escape rooms differ in their goals. Whilst recreational escape rooms are developed to enjoy participations in a playful informal learning environment, educational escape rooms are developed to stimulate the formal learning process of students on a specific content.^20^, ^21^ This difference in main goal between a recreational and educational escape room leads to important design criterium for educational escape rooms namely that quizzes and puzzles in the escape room should be in line with the learning goals.

In addition to the constructive alignment between learning goals and quizzes and puzzles, an effective educational escape room implements at least the three elements; immersion, collaboration, and debriefing. These design elements were also present in the pharmacokinetics escape room. Immersion was done by either a telephone call (lab group) or the video on a USB stick (office group). Moreover, students had to collaborate within and between the two groups and there was a debriefing directly after the game. Recently, it became known that immersion relates directly to the knowledge gain in students as was observed in grade 10–12 high‐potential students, aged 16–20 years.^20^

Despite other research showing that escape rooms are effective teaching and learning methods,^20^, ^21^, ^22^ students in this study doubted the usefulness of the escape room as a formative test. This discrepancy in outcome might be explained by the difference between the final test questions on the pharmacokinetics exam and the puzzles and quizzes students encountered in the escape room. The final exam consisted mainly of open‐ended, short‐essay questions while puzzles and quizzes in the escape room were more closed and/or multiple‐choice type questions as these latter types can be easily converted into codes for opening boxes or getting computer access. Another explanation for the discrepancy in results could be the difference in the difficulty of the puzzles and quizzes and the open‐ended short‐essay questions. The difficulty in teaching and learning outcomes can be determined by hierarchal classifications of the different levels of thinking ranging from low‐order toward higher‐order thinking, like Bloom's taxonomy or Biggs' SOLO taxonomy.^23^, ^24^ While the open‐ended short‐essay questions consisted of both lower‐order thinking questions and higher‐order thinking questions, the puzzles and quizzes in the escape room were primarily of lower‐order thinking.

Certain limitations should be considered. First, data were limited in number and from a single course. Student enrolment in the course is limited due to the selective nature of the undergraduate program but why not more students, especially in the control group, completed the inventory is unknown. Conducting interviews could have given more insight in students' motivation to join this study as well as more insight in the limited effects of the escape room on the MUSIC® subscales other than situational interest. Second, this study was conducted while the pharmacology course was being redesigned. The redesign was to improve the inquiry‐based learning format of the course by implementing self‐study modules. Redesign of the course as well as the selective nature of the program could possibly explain the high score on caring and the absence of effects on the scales other than situational interest.

In conclusion, the results of this study demonstrated both quantitatively and qualitatively that playing a pharmacokinetics escape room increased students' perceived situational interest. Since situational interest is one of the key features of motivation, this suggests that playing a general pharmacokinetics‐based escape room can enhance students' motivation in a course on general pharmacology. Our findings add to the literature that game‐based learning has a positive effect on students' motivation and engagement. Further research should focus on the effect of the escape room on academic success (e.g., grades of the pharmacokinetics exam) and preferably include a sizeable control group as well as upscaling the capacity of the escape room for large‐scale courses.

## AUTHOR CONTRIBUTIONS

Freija ter Heegde, Anneke van Houwelingen, and Theo Wubbels designed the study; Freija ter Heegde, Wendy Boschloo, Leonie Piek, and Anneke van Houwelingen designed the escape room; Freija ter Heegde acquired the data; Anneke van Houwelingen and Theo Wubbels performed the quantitative statistical analysis; AvH performed the qualitative analysis; Freija ter Heegde and v wrote the paper. All authors have commented, revised, and approved the final version of the paper.

## FUNDING INFORMATION

This project was supported by the Utrecht University Education Incentive Fund.

## CONFLICT OF INTEREST STATEMENT

No conflicts of interest.

## ETHICS STATEMENT

All participants in this study gave their consent at the beginning of the questionnaire. Ethical approval for this study was obtained from the Science‐Geosciences Ethics Review Board Committee of the Utrecht University (Bèta S‐21653).

## Data Availability

The data that support the finding of this study are available from the corresponding author upon reasonable request.
